# A delicate balance: role of MMP-9 in brain development and pathophysiology of neurodevelopmental disorders

**DOI:** 10.3389/fncel.2015.00280

**Published:** 2015-07-29

**Authors:** Sarah M. Reinhard, Khaleel Razak, Iryna M. Ethell

**Affiliations:** ^1^Psychology Department, University of California, RiversideRiverside, CA, USA; ^2^Biomedical Sciences Division, School of Medicine, University of California, RiversideRiverside, CA, USA

**Keywords:** matrix metalloproteinase-9, critical period, plasticity, neurodevelopment, extracellular matrix

## Abstract

The extracellular matrix (ECM) is a critical regulator of neural network development and plasticity. As neuronal circuits develop, the ECM stabilizes synaptic contacts, while its cleavage has both permissive and active roles in the regulation of plasticity. Matrix metalloproteinase 9 (MMP-9) is a member of a large family of zinc-dependent endopeptidases that can cleave ECM and several cell surface receptors allowing for synaptic and circuit level reorganization. It is becoming increasingly clear that the regulated activity of MMP-9 is critical for central nervous system (CNS) development. In particular, MMP-9 has a role in the development of sensory circuits during early postnatal periods, called ‘critical periods.’ MMP-9 can regulate sensory-mediated, local circuit reorganization through its ability to control synaptogenesis, axonal pathfinding and myelination. Although activity-dependent activation of MMP-9 at specific synapses plays an important role in multiple plasticity mechanisms throughout the CNS, misregulated activation of the enzyme is implicated in a number of neurodegenerative disorders, including traumatic brain injury, multiple sclerosis, and Alzheimer’s disease. Growing evidence also suggests a role for MMP-9 in the pathophysiology of neurodevelopmental disorders including Fragile X Syndrome. This review outlines the various actions of MMP-9 during postnatal brain development, critical for future studies exploring novel therapeutic strategies for neurodevelopmental disorders.

## Introduction

The capacity of the CNS of an animal to adapt to changing internal and external environments must be offset by the capacity to maintain network stability following changes. This stability-plasticity balance is highly regulated and is necessary for adaptation and survival throughout the lifespan of an organism. An important challenge in neuroscience remains to identify the mechanisms that regulate the dynamics between stability and plasticity. With some 86–100 billion neurons and an approximately equal or larger number of glial cells, depending upon the brain region (Azevedo et al., 2009), the study of the brain has been likened to the quest for understanding the universe, with multiple cellular and molecular interactions that account for brain plasticity. Yet many key mechanisms underlying synaptic plasticity are found not in these brain cells themselves but in their interactions with molecules in the extracellular space surrounding them (reviewed by Ruoslahti, 1996a,b; Nicholson and Sykova, 1998; Shi and Ethell, 2006; Šišková et al., 2009; Dityatev et al., 2010; Dansie and Ethell, 2011). The ECM can be thought of as a scaffold that surrounds neurons and glia within the extracellular space. It is rich with signaling cues that can guide plasticity while maintaining stable network connections over time, mediating synaptic and trans-synaptic interactions. These components can act as repulsive and/or attractive cues for cellular migration and can mediate cell-ECM or cell–cell interactions. While the role of ECM in the plasticity of neuronal circuits is not yet well understood, cleavage of the ECM through the actions of matrix metalloproteinases (MMP) and other ECM-cleaving enzymes can drive plasticity in response to specific changes in neuronal activity.

Matrix metalloproteinase-9 in particular stands out as an important molecule in CNS development and plasticity. MMP-9 is a Zn^2+^dependent endopeptidase expressed in both the central- and peripheral nervous systems and acts to cleave components of the ECM as well as cell adhesion molecules, cell surface receptors and other proteases. Research has focused on the role of MMP-9 in the progression of neurologic disorders such as epilepsy, multiple sclerosis, neuroinflammatory and autoimmune disorders. However, converging evidence suggests that MMP-9 plays an important role in both establishing synaptic connections during development and in the restructuring of synaptic networks in the adult brain. As improper maturation of sensory networks during development is implicated in many neurodevelopmental disorders and in cognitive deficits, understanding the mechanisms of MMP-9 mediated synaptic plasticity is essential for the development of therapeutic strategies. Such knowledge will guide future clinical studies on the possible role of MMP-9 in neurodevelopmental disorders. As it is possible to regain a state of plasticity and reverse cognitive deficits by manipulating the onset and closure of ‘critical periods’ (Bradbury et al., 2002; Pizzorusso et al., 2002, 2006), regulating MMP-9 activity during CPP may aid in further development of precise and targeted treatments for neurodevelopmental disorders.

## MMP-9 Activity, Expression and Regulation

Matrix metalloproteinase-9 is a Zn^2+^ dependent endopeptidase that is found in many cell types throughout the body, including neurons and glia, endothelial cells (Genersch et al., 2000), glandular epithelia, supportive connective tissue, and muscle cells (Roomi et al., 2009). Among the 25 known MMPs (reviewed by Ethell and Ethell, 2007), MMP-9, MMP-2 and MMP-3 are widely expressed in the CNS. Furthermore, MMP-9 and MMP-2 share similar substrate specificity and are known as gelatinases. Their substrates include components of the ECM, as well as cell adhesion molecules and cell surface receptors, cytokines, growth factors, and other proteases (reviewed by Ethell and Ethell, 2007). Beside an active site containing Zn^2+^ that is necessary for its enzymatic activity, MMP-9 also contains three fibronectin type II repeats which can directly bind gelatin, laminin and collagens types I and IV (reviewed by Van den Steen et al., 2002).

Matrix metalloproteinase-9 expression is regulated during development, with high levels during early developmental time-points that decrease into adulthood (Vaillant et al., 1999; Oliveira-Silva et al., 2007; Bednarek et al., 2009; Aujla and Huntley, 2014). This pattern is consistent when measuring either the protein levels of MMP-9 with antibodies or the enzymatic activity levels of MMP-9 using gelatin zymography (Oliveira-Silva et al., 2007). Although MMP-9 levels remain low in the adult brain, MMP-9 activity increases in response to synaptic activity (Gawlak et al., 2009; Janusz et al., 2013). MMP-9 expression has been detected in several brain areas, including the hippocampus (Aujla and Huntley, 2014), the brainstem (Oliveira-Silva et al., 2007), the cerebellum (Vaillant et al., 1999), and the neocortex (Bednarek et al., 2009). MMP-9 proteolytic activity co-localizes with excitatory synapses (Gawlak et al., 2009), while it’s mRNA is detected within the cell body, along the processes and in synaptoneurosome fractions of neurons (Janusz et al., 2013). MMP-9 is also expressed in astrocytes, microglia and along oligodendrocyte processes (reviewed by Dzwonek et al., 2004; Gawlak et al., 2009).

Importantly, the expression, translation and activity of MMP-9 are tightly regulated. Expression of MMP-9 can be regulated by growth factors, cytokines, oncogenes, metal ions, and hormones through MAPK pathway signaling (reviewed by Van den Steen et al., 2002). MMP-9 translation is suppressed through its binding to FMRP, an mRNA binding protein, implicating MMP-9 in fragile X syndrome (FXS) (Janusz et al., 2013). MMP-9 mRNA is mainly localized in the cell body of hippocampal neurons and is translocated from the cell body to dendritic synapses in response to neuronal activity (Dziembowska et al., 2012) as a part of an FMRP containing granule (Janusz et al., 2013). mGluR activation causes the dissociation of FMRP from the MMP-9 mRNA (Janusz et al., 2013) followed by the association of MMP-9 mRNA with actively translating polyribosomes and *de novo* protein synthesis (Dziembowska et al., 2012). Once the MMP-9 protein is secreted from a cell by a yet to be discovered mechanism, it is in an inactive pro-from, also called a zymogen, where its enzymatic activity is inhibited by a pro-domain that masks the catalytic site through an interaction between Zn^2+^ and a cysteine residue in the pro-domain (reviewed by Ethell and Ethell, 2007). This pro-form cannot cleave its substrates until the pro-domain has been removed from the active site by proteolysis or protein unfolding, a process called a *cysteine switch*. This can be performed by other MMPs, serine proteinases, by nitric oxide and by reactive oxygen species. The action of MMP-9 is further limited by degradation of the enzyme and by inhibition of its activity via thrombospondins or through tissue inhibitor of metalloproteinase-1 (TIMP-1) – which interestingly is secreted in response to synaptic activity at levels similar to MMP-9 (reviewed by Ethell and Ethell, 2007) and can form a complex with MMP-9 prior to secretion (Roderfeld et al., 2007). Thus, the tight regulation of MMP-9 activity is critical for its function in development and plasticity, and highlights a role for MMP-9 in cell-specific, activity-driven activation and remodeling of the pericellular environment, with spatially and temporally restrained effects.

A large body of research on MMP-9 has focused on its various roles in neurodegenerative disorders (reviewed by Yong, 2005). Nonetheless, it is becoming increasingly clear that MMP-9 has multiple functions in CNS development as well, and may contribute to neurodevelopmental disorders. MMP-9, through cleavage of surface- and cell-adhesion molecules, is implicated in active dendritic spine remodeling and stabilization (reviewed by Bilousova et al., 2009; Benson and Huntley, 2012), affecting the shape and function of dendritic synapses (Michaluk et al., 2011). MMP-9 also plays a role in pre- and postsynaptic receptor dynamics (Michaluk et al., 2009; Peixoto et al., 2012; Ning et al., 2013), in consolidation of long-term potentiation (LTP; Wang et al., 2008), myelination (Oh et al., 1999), and possibly in synaptic pruning (Wilczynski et al., 2008). It is further implicated in axonal elongation (Shubayev and Myers, 2004), pathfinding (Vaillant et al., 2003; Lin et al., 2008; Aujla and Huntley, 2014), regeneration (Ahmed et al., 2005) and degeneration (Costanzo et al., 2006). This review summarizes the various roles of MMP-9 in diverse developmental processes within the CNS, with an emphasis on sensory development, and suggests a role for misregulated MMP-9 in neurodevelopmental disorders.

## Brain Development and Critical Period Plasticity

The critical period can be loosely defined as a time when experience-dependent activity can drive alterations in the structure, functional organization and physiological properties of brain regions. It occurs during postnatal development and is marked by increased plasticity and massive synaptic reorganization. The opening of the critical period is driven by the onset of sensory function, when peripheral sensory structures mature and begin to relay external sensory input into central structures. Reorganization takes place among thalamocortical projections, established prior to sensory input (reviewed by Feller and Scanziani, 2005), and among corticocortical connections, which together undergo increased synaptogenesis, axonal arborization, and pruning (Sanes et al., 2005) so that each sensory modality is able to respond optimally to behaviorally relevant stimuli (reviewed by Hensch, 2004). This increased plasticity during the critical period is time-limited, constrained by the development of stabilizing factors such as increased intracortical inhibition and the formation of PNNs, a specialized ECM structure (reviewed by Hensch, 2005). The timing of this plasticity is different depending on species, sensory modality and even on the type of sensory manipulation used within a sensory modality (reviewed by Hensch, 2004). Non-sensory regions likewise have a window of postnatal synaptogenesis, reorganization, pruning and apoptosis (reviewed by Hashimoto and Kano, 2005; reviewed by Ribak et al., 1985; Steward and Falk, 1991; Luo, 2005; Murase and McKay, 2012). Periods of high MMP-9 activity correlate with synaptic reorganization during CPP and developmental windows across CNS regions (see **Table 1**), suggesting a role for MMP-9 in shaping CPP kinetics. Within this review we discuss CPP within rodent models due to the widespread use of these models for genetic manipulation of MMP-9 activity, and note that time-lines provided in this review for CPP windows are subject to variability between experimental procedures, protocols and outcome measures used, and are intended as a basic guideline.

**Table 1:** Matrix metalloproteinase-9 expression in CNS development.

Brain region	Developmental window	Peak of MMP-9 expression	MMP-9 in experience dependent plasticity	Reference
Retinotectal Axons Visual Cortex Optic Nerve	Retinotectal map formation: P0–P21 (first 3 postnatal weeks) *r.* Monocular deprivation: P21–P28 *r.*; Postnatal week 3–5 *r.;* P19–P32 *m.* Optic nerve myelination: P7–P9 *m.*	Sup. Colliculus: P0–P10 *protein* P0–P14 *activity.* Optic Nerve: P7-P11 *activity.* P7 *mRNA*	Monocular enucleation increases MMP-9 enzymatic activity in SC Monocular deprivation increases MMP-9 mRNA in cortex	Oliveira-Silva et al. (2007) Fagiolini et al. (1994), Gordon and Stryker (1996), Spolidoro et al. (2012) Oh et al. (1999), Larsen et al. (2006)
Barrel Cortex	Thalamocortical input to Layer 4: P1-P4/5 *m., r.* Intracortical connections: Layer 4-2/3: P10-P14 *r.* Layer 2/3: thru adulthood *r.*		Whisker trimming in adolescent mice increases MMP-9 enzymatic activity; MMP-9 KO mice show reduced potentiation in L4 and L2/3 following trimming	Woolsey and Wann (1976), Kossut et al. (1988), Fox (1992), Maravall (2004), Kaliszewska et al. (2012)
Cerebellum	Granule cell proliferation, Climbing fibers refinement: Postnatal week 1 Granule cell migration; Parallel fiber innervation of Purkinje cells: Postnatal week 2–3 Purkinje cell arborization: Postnatal week 4	Granule precursors, Bergmann glia, Purkinje cells: P10 *protein* EGL, IGL and Purkinje cell layer: P11-P17 *protein*	MMP-9 KO mice at P12 exhibit: Decreased apoptosis of granule precursor cells; Delay in granule cell migration	*r.* Altman (1972a,b), *r*. Vaillant et al. (1999), *r.* Piccolini et al. (2012), *m*. Vaillant et al. (2003)
Hippocampus	Spontaneous activity and apoptosis: P0–P7 *r. m.* Mossy fiber development: first postnatal month *r.* Synapse elaboration: first postnatal month *r.*	Axon tips: P4 *r. protein* DG, CA1, CA3: P4 r. *mRNA* Postsynaptic densities: P8 *r. protein*	MMP-9 mediates cell survival through interactions with laminin MMP-9 KO mice show accelerated synaptic maturation MMP-9 treatment exaggerates immature spine morphology	Ribak et al. (1985), Steward and Falk (1991), Bilousova et al. (2009), Murase and McKay (2012), Aujla and Huntley (2014), Sidhu et al. (2014)
Auditory Cortex	Tonotopic reorganization: P11–P15 *m*. P11–P13 *r.* P9–P20 *m.* P11–P28 *r.*		Cochleotomy leads to increased MMP-9 levels in cochlear nucleus 1 day post-op	Zhang et al. (2002), de Villers-Sidani et al. (2007), Illing et al. (2010), Barkat et al. (2011), Kim et al. (2013)

*r., rats; m., mouse; activity, enzymatic activity measure by zymography.*

One of the first demonstrations of CPP came from studies by Hubel and Wiesel (1964) in the kitten visual cortex, where they demonstrated that monocular deprivation alters both functional and anatomical organization in the cortex and, to a more limited extent, in the thalamus. Within the rodent visual cortex, the CPP window opens during the third postnatal week and closes into the fifth postnatal week, with adult-like functional properties by P45 (Fagiolini et al., 1994; Gordon and Stryker, 1996), though retinotectal reorganization occurs mostly in the first three postnatal weeks (Serfaty et al., 2005; Oliveira-Silva et al., 2007). Most neurons in the rodent visual cortex (Sawtell et al., 2003) and SC respond to contralateral eye stimulation and this contralateral preference can be manipulated during development by monocular deprivation. After eye closure (monocular deprivation), there is a reorganization of network connections contralateral to the deprived eye within the visual cortex (Spolidoro et al., 2012) and SC (Oliveira-Silva et al., 2007). Neuronal responses to stimulation of the deprived eye undergo depression and weakening of synaptic efficacy within 3 days (Sawtell et al., 2003), along with shrinkage of thalamocortical projections (reviewed by Feller and Scanziani, 2005; Spolidoro et al., 2012). Between 4 and 7 days after monocular deprivation, responses to the non-deprived eye undergo potentiation, with a concomitant expansion in axonal arborization (reviewed by Sawtell et al., 2003; Feller and Scanziani, 2005; Spolidoro et al., 2012). The overall outcome is that more neurons will respond to visual stimulation of the non-deprived ipsilateral eye, termed an ocular dominance shift.

Matrix metalloproteinase-9 has been implicated in various aspects of visual CPP. In the SC, MMP-9 enzymatic activity and protein levels (both the proform and active forms) are high during the period of retinotectal map reorganization before and just after eye opening (from P0 to P14), and decrease during late (P21–P42) postnatal development (Oliveira-Silva et al., 2007). Broad inhibition of MMPs from P0 to P7 alters the topographical organization of retinotectal terminals, while monocular enucleation at P10 increases MMP-9 enzymatic activity in the SC both contralateral and ipsilateral to the enucleated eye (Oliveira-Silva et al., 2007). Within the cortex, MMPs are involved in plasticity following monocular deprivation for either 3 or 7 days in adolescent rats (P21–P45; Spolidoro et al., 2012). Inhibition of MMP activity, using the broad spectrum MMP inhibitor GM6001 produces three main effects: (1) reduces the ocular dominance shift after monocular deprivation by selectively preventing the potentiation of open-eye responses without affecting the depression of deprived-eye responses, (2) blocks the deprivation-driven increase in spine density in layer 2/3 excitatory neurons contralateral to the deprived eye, and (3) blocks the potentiation of the deprived-eye responses after eye reopening. Though MMP-9 cannot be directly implicated in cortical ocular dominance plasticity based on these results, the authors noted that the only MMP to show mRNA changes in response to monocular deprivation was MMP-9 (Spolidoro et al., 2012). MMP-2 is, however, expressed in the CNS at higher basal levels than MMP-9, and therefore may account for ocular dominance plasticity without mRNA up-regulation. To our knowledge there is no published work using MMP-9 KO mice to further test the role of MMP-9 in visual CPP, however, MMP-3 KO mice, which have reduced activation of pro-MMP-9, do demonstrate altered visual CPP plasticity including reduced open-eye potentiation, suggesting a possible role for MMP-9 (Aerts et al., 2014).

Matrix metalloproteinase-9 has been more directly implicated in somatosensory barrel cortex plasticity. In rodent barrel cortex each whisker is represented in a one-to-one ratio by a cortical cellular aggregate, termed a ‘barrel.’ Both the thalamocortical innervation of the barrels as well as the intracortical connections can be modified by removing the whiskers, with less dramatic reorganization after plucking or trimming of the whiskers. There are multiple windows of plasticity between cortical layers and across ages (reviewed by Fox, 2002), and the effect of experimental manipulation on barrel cortex plasticity depends both on the age, length and the type of manipulation used. Early postnatal CPP takes place largely in layer IV barrel cortex from P0 to P4/5 (Woolsey and Wann, 1976; Fox, 1992). It is characterized by receptive field plasticity, a conversion of silent synapses into active synapses, and changes in pre-synaptic thalamocortical arborization and barrel formation (reviewed by Fox, 2002 and Erzurumlu and Gaspar, 2012). Lesions within this window can induce structural reorganization of thalamocortical afferents (reviewed by Fox, 2002; Erzurumlu and Gaspar, 2012). CPP in layer IV to II/III occurs from P10 to P14 (reviewed by Maravall, 2004; Erzurumlu and Gaspar, 2012), whereas horizontal intracortical connections in layers II/III remain plastic through the third postnatal week and, to a lesser extent, throughout adolescence (reviewed by Fox, 2002). Adolescent plasticity is characterized as functional but not structural reorganization, including potentiation of spared input and depression of deprived input. More limited functional plasticity remains into adulthood (reviewed by Kossut et al., 1988; Fox, 2002).

One common manipulation to examine barrel CPP is to trim all but one row of whiskers. This can cause a topographic reorganization of functional sensory maps so that barrel columns in the cortex that have been deprived of peripheral sensory input will respond to deflections of the retained, intact whiskers. Such reorganization is possible in adolescent mice after 1 week of whisker trimming, and even more pronounced after 4 weeks. This manipulation causes an increase in the width of barrel activation when the spared-whiskers are deflected, an effect seen in all cortical layers of a vertical barrel column (Kaliszewska et al., 2012). When the spared-whisker is deflected there is also increased cellular activation in the barrel columns that directly border the spared row, as measured by c-fos density (Kaliszewska et al., 2012). After undergoing this deprivation-induced plasticity for 1 week, MMP-9 enzymatic activity levels are higher in the barrel cortex of mice when compared to the control non-deprived hemisphere, whereas there is no increase in MMP-2 (Kaliszewska et al., 2012). In MMP-9 KO mice, though 4 weeks of deprivation did increase the width of spared-row activation in all cortical layers when compared to the control non-deprived hemisphere, the width of layer IV activation was significantly smaller than in WT mice (Kaliszewska et al., 2012). Cellular activation of the deprived rows was also significantly reduced in layer II/III of MMP-9 KO mice compared to WT mice, but was no different in any other layer (Kaliszewska et al., 2012). The fact that MMP-9 activity did not affect reorganization at all layers indicates a specific action of MMP-9, though the mechanism of the specificity is not known.

Understanding how MMP-9 is affecting plasticity at only certain levels of the circuit is difficult based on these results. The most likely site of functional reorganization in adult barrel cortex after deprivation or denervation may be through the unmasking of previously established silent or ‘weak’ synapses that project from the intact whisker onto neighboring columns (Simons, 1978; Kossut et al., 1988). It is possible that MMP-9 is necessary for integrating these weaker synapses into the circuit by maturation and stabilization of synaptic contacts (see Synapse Development and Plasticity below). Conversely, increased responses to the intact whisker column may be due to disinhibition from surrounding barrels (Kelly et al., 1999). The decreased potentiation in layer II/III observed in the deprived neighboring barrel columns in MMP-9 KO mice may be an effect of the reduced potentiation in layer IV of the intact barrel, as measurement of response latency suggests that information is transferred within a barrel column first, beginning in layer IV and transferring vertically between layers, before it is transferred outward to neighboring barrels (Armstrong-James et al., 1992; reviewed by Fox, 2002; Feldmeyer, 2012). It is further possible that the increased functional activation in layer IV was reflective of changes in astrocytic activity instead of or in addition to neuronal activity. The method of 2-DG autoradiography used in Kaliszewska et al. (2012) measures metabolic activity including that of astrocytes which are located within the somatosensory barrel cortex and which show an organizational pattern that parallels neuronal organization (Giaume et al., 2009; Logvinov et al., 2011). It is possible that loss of MMP-9 can affect astrocytic function and that as-yet undetermined interactions exist between astrocytes, MMP-9 activity and synaptic plasticity in layer IV barrel cortex. More studies are needed to clarify the mechanisms that underlie changes in plasticity observed here.

To our knowledge, no studies have looked at the role of MMP-9 in auditory system CPP. MMP-9 does not influence the development of spiral ganglion cells, which relay information from cochlear hair cells to the CNS (Sung et al., 2014). On the other hand, after removal of the cochlea and spiral ganglion cells, MMP-9 protein in the cochlear nucleus peaks 1 day post-operation, with an increase in the neuropil and a decrease within cell bodies, suggestive of increased MMP-9 secretion from the cells and indicating that MMP-9 may be involved in degenerative and/or regenerative processes in early auditory pathways (Illing et al., 2010). Indirect evidence suggests MMP-9 may have a role in auditory CPP. In *Fmr1* KO mice, a mouse model of FXS, auditory cortex CPP is deficient (Kim et al., 2013). MMP-9 enzymatic levels are elevated in the brain of *Fmr1* KO mice (Gkogkas et al., 2014; Sidhu et al., 2014) possibly due to a loss of FMRP transcriptional regulation of MMP-9 (Janusz et al., 2013). Genetic deletion of MMP-9 or pharmacological treatment that lowers MMP-9 levels during postnatal development rescues behavioral deficits in *Fmr1* KO mice in adulthood (Dansie et al., 2013; Sidhu et al., 2014). Therefore, it is possible that elevated MMP-9 underlies deficient auditory cortex CPP in the *Fmr1* KO mice, which remains to be tested. This is an important avenue of research because CPP deficits may underlie later auditory processing deficits in FXS patients (Rotschafer and Razak, 2013; reviewed by Rotschafer and Razak, 2014).

In the rodent cerebellum, a peak in MMP-9 expression also correlates with a window of synaptic and cellular reorganization. The cerebellum has a clear laminar distribution of cell types and function, with a deep layer called the granular layer or internal granular layer (IGL), a middle layer called the Purkinje cell layer, and a superficial layer called the molecular layer. During early postnatal development there is yet another layer which covers the surface of the cerebellum, the external granular layer (EGL). Cerebellar postnatal development is marked first by the proliferation of cerebellar granule neuron precursor cells in the EGL during the first postnatal week (Altman, 1972a; reviewed by Luo, 2005) and a refinement of climbing fiber excitatory input onto Purkinje cells (reviewed by Hashimoto and Kano, 2005). During the second postnatal week the EGL thins following the migration of granule precursor cells (Vaillant et al., 1999) and disappears by the end of the third postnatal week. During this same time frame the refinement of climbing fiber connections onto Purkinje cells is followed by the innervation of parallel fibers (granule cell axons) onto Purkinje cells as the Purkinje cells begin to form secondary and tertiary dendritic branches (Altman, 1972b; reviewed by Hashimoto and Kano, 2005). Finally Purkinje cells undergo an extensive arborization during the fourth postnatal week (reviewed by Luo, 2005). MMP-9 enzymatic activity peaks at P10 and decreases to adult levels by P15 (Vaillant et al., 1999). MMP-9 protein at P10 is detectable in granule precursor cells, Bergmann glial cell bodies and processes, and in Purkinje cell dendrites, a pattern which is similar to MMP-9 mRNA expression (Vaillant et al., 1999). From P11 to P17, both MMP-9 and MMP-3 protein are expressed in the EGL, the IGL, and intensely in the molecular and Purkinje cell layers (Piccolini et al., 2012) where MMP-9 labels the soma of granule and Purkinje cells (Vaillant et al., 2003). At P12 MMP-9 expression in the EGL is concentrated in the premigratory pool of granule precursor cells and is present in migrating granule precursor cells dispersed in the molecular layer (Vaillant et al., 2003). Overlapping expression of MMP-9 and MMP-3 is important because MMP-3 can cleave and activate MMP-9. Indeed, MMP-9 plays an important role in cerebellar development as the application of an MMP-9 blocking antibody and genetic deletion of MMP-9 inhibit axonal outgrowth (parallel fibers) and the migration and apoptosis of granule cell precursors in the developing cerebellum (Vaillant et al., 2003).

In the hippocampus, structural organization is largely established during embryogenesis, though in the germinal zone of the DG active neurogenesis and granule cell migration continue into adulthood (Bayer, 1980). During the first postnatal week, there is spontaneous activity that drives reorganization and neuronal survival (Murase and McKay, 2012), while mossy fibers (Ribak et al., 1985) as well as synapses within the DG and CA1 (Steward and Falk, 1991) undergo elaboration and maturation throughout the first postnatal month. At P4, MMP-9 mRNA levels peak in the DG, CA1 and CA3, and MMP-9 and MMP-2 proteins are shown to co-immunolabel with L1CAM (L1 cell adhesion molecule), a marker of growing axons (Aujla and Huntley, 2014). At a period of peak hippocampal synaptogenesis, MMP-9 and MMP-2 are localized near the postsynaptic densities immunolableled against PSD-95 (Aujla and Huntley, 2014). MMP-9 genetic deletion accelerates the maturation of dendritic spines showing an early increase in the number of mature mushroom-shaped spines at P8, which is normally observed later at P14–P21 (Sidhu et al., 2014). Conversely, an acute treatment of cultured hippocampal neurons with active MMP-9 leads to an increase in immature filopodia-like spines and a decrease in mature mushroom-shaped spines (Bilousova et al., 2009). While global genetic deletion of MMP-9 causes an increase in the number of mature spines during early postnatal hippocampal development and enhanced MMP-9 activity leads to the formation of immature spines in cultured hippocampal neurons, a transient release of MMP-9 at the level of a single synapse was shown to be associated with LTP-induced spine enlargement in adult hippocampus (Wang et al., 2008; see Synapse Development and Plasticity for more details). MMP-9 has been also implicated in spine head maturation during the development of visual cortex (Kelly et al., 2014). It is possible that genetic deletion of MMP-9 can lead to brain area specific compensatory effects of other MMPs (Esparza, 2004; Nagy et al., 2006), as MMP-3 is also expressed in the brain and is implicated in synaptic plasticity (Aerts et al., 2014).

Taken together, these studies suggest MMP-9 activity contributes to multiple aspects of neural network reorganization during early development, including regulation of CPP plasticity. This sensory-dependent, activity driven reorganization primes each region to respond appropriately to external cues. The mechanisms underlying MMP-9-mediated CPP and synaptic reorganization though, are not well understood (see **Table 2**).

**Table 2:** Matrix metalloproteinase-9 role in specific plastic events.

		Reference
**Synaptic Plasticity**		
	Late-phase LTP	Nagy et al. (2006), Okulski et al. (2007), Wang et al. (2008), Wiera et al. (2013)
	NMDA receptor trafficking	Michaluk et al. (2009)
	AMPA receptor trafficking	Szepesi et al. (2014)
	Spine elongation	Bilousova et al. (2009), Michaluk et al. (2009, 2011)
	Spine maturation	Wang et al. (2008), Kelly et al. (2014), Szepesi et al. (2014)
	Integrin-dependent	Wang et al. (2008), Michaluk et al. (2009, 2011)
	Involves ICAM-5	Tian et al. (2007), Ning et al. (2013), Kelly et al. (2014)
**Axonal plasticity**		
	Pathfinding	Vaillant et al. (2003), Hehr (2005), Aujla and Huntley (2014)
	Regeneration after injury	Ferguson (2000), Shubayev and Myers (2004), Ahmed et al. (2005)
	Degeneration after injury	Costanzo et al. (2006)
	EphB2 mediated growth cone collapse	Lin et al. (2008)
**Myelination**		
	Oligodendrocyte process extension/formation	Uhm et al. (1998), Oh et al. (1999), Šišková et al. (2009)
	Oligodendrocyte/axon contact	Maier et al. (2006)

## Cellular Mechanisms

### Synapse Development and Plasticity

The structural changes within the CNS thought to underlie neural plasticity, including learning and memory, can be traced to the level of the individual synapse and spine. Functional synaptic plasticity can be broadly classified into two underlying processes: one being depression, during which the post-synaptic potentials are reduced in amplitude relative to baseline, and the other being potentiation, characterized as an increase in synaptic strength. Additionally these changes in synaptic efficacy can be transient (short-term) or they can be maintained hours to days (long-term), where modification of receptors at synaptic sites through new protein synthesis is thought to underlie long-term changes. The mechanisms of both potentiation and depression can be localized to the presynaptic axon terminal, the postsynaptic dendrite, or both. Synaptic plasticity is a process that can incorporate new or changing input into a system, can form associative networks of connections and can respond to changes in the nervous system environment by scaling connections up or down over time to allow for fine-tuned network modification. As such, synaptic plasticity can be considered to be a fundamental cellular event underlying much of the ability of the brain to adapt and respond to changing environmental demands throughout a lifetime, from CPP sensory reorganization to learning and memory.

Matrix metalloproteinase-9 has been implicated in the maintenance of LTP. LTP can be classified into an early-phase LTP (e-LTP) that is independent of protein synthesis and a late-phase LTP (l-LTP) that requires new protein synthesis. l-LTP in particular may underlie the consolidation of memories by strengthening synapses for extended periods of time. A popular brain structure for the study of synaptic plasticity mechanisms is the hippocampus, due to its well-defined circuit organization and because of its role in learning and memory. Multiple hippocampal pathways have been used to study the role of MMP-9 in synaptic plasticity. In the mossy fiber-CA3 pathway, LTP induction in adolescent rats causes an increase in MMP-9 enzymatic activity, both the proform and the active form of the enzyme, and an increase in MMP-9 protein expression in CA3 neurons (Wiera et al., 2012). Both total genetic deletion of MMP-9 (mice) and a 15-fold overexpression of MMP-9 (rats) impair the maintenance of LTP in the mossy fiber-CA3 pathway in hippocampal adolescent slices (Wiera et al., 2013). In both MMP-9 KO and overexpressing models the initial potentiation after high frequency tetanic stimulation is comparable to wild types, but as early as 7–10 min post-stimulation, both models show a significantly reduced potentiation (Wiera et al., 2013). This indicates that too much or too little MMP-9 can lead to impaired LTP maintenance, including both early- and late-phase LTP. In a different hippocampal pathway, the Schaffer collateral-CA1 pathway, MMP-9 activity is also implicated in LTP. High frequency tetanic stimulation or chemically induced LTP (cLTP) increase the levels of active MMP-9 protein, peaking 30 min post-stimulation, but do not affect the levels of MMP-2 (Nagy et al., 2006). cLTP also increases the proteolytic activity of MMP-9 localized to the neuropil in CA1 stratum radiatum (Nagy et al., 2006). Inhibition of MMP-9 and MMP-2 in slices of both young and adult rats, or inhibition of MMP-9 alone in anesthetized rats *in vivo* (Bozdagi et al., 2007), does not affect initial potentiation and e-LTP but does impair l-LTP, with decreased potentiation by 60 min post-stimulation (Nagy et al., 2006). Total genetic deletion of MMP-9 in this same pathway impairs LTP overall, similar to the mossy fiber-CA3 pathway (Nagy et al., 2006). In the CA1 to prefrontal cortex pathway TIMP-1 overexpression, which reduces MMP activity, prevents l-LTP *in vivo* (Okulski et al., 2007). Similarly direct inhibition of MMP-9 *in vitro* blocks prefrontal l-LTP but not e-LTP (Okulski et al., 2007). The precise mechanisms by which MMP-9 affects synaptic plasticity are not fully understood. In the Schaffer collateral-CA1 pathway, increased MMP-9 levels after tetanic stimulation were dependent on *N*-methyl-D-aspartate (NMDA) receptor activation at postsynaptic sites (Nagy et al., 2006). Conversely in the mossy fiber-CA3 pathway, where LTP is also MMP-9 dependent, LTP occurs at presynaptic sites and is independent of NMDA receptors (Harris and Cotman, 1986; Castillo et al., 1997; Wiera et al., 2013). Moreover the experimental manipulations used to test MMP-9 necessity, by either genetic deletion or chemical inhibition, have differential effects on e-LTP induction, leaving in question the role of MMP-9 in e-LTP.

Matrix metalloproteinase-9 also affects the trafficking of both NMDA and α-amino-3-hydroxyl-5-methyl-4-isoxazole-propionate (AMPA) glutamate receptors. The modification and/or insertion of glutamate receptors to the postsynaptic site are thought to fundamentally relate to long-term synaptic plasticity. Bath application of auto activating MMP-9 in cultured hippocampal neurons induces the lateral diffusion of NMDA receptors, but not GluA2-containing AMPA receptors, at synapses (Michaluk et al., 2009). On the other hand, after a chemical LTP (cLTP) induction in cultured hippocampal neurons, MMP activity is necessary for the immobilization and synaptic accumulation of GluA1- and GluA2-containing AMPA receptors (Szepesi et al., 2013, 2014) in dendritic spines. MMP-9 in particular was suggested to be involved in AMPA receptor trafficking because MMP-9 levels, but not MMP-2, were up-regulated following cLTP (Szepesi et al., 2013). The action of MMP-9 on lateral diffusion of NMDA receptors was shown to depend on integrin β1 signaling (Michaluk et al., 2009). Integrins are membrane-bound ECM receptors found on pre-and postsynaptic sites in many types of cells in the CNS. Activation of integrin receptors induces the elongation of dendritic spines in hippocampal culture (Shi and Ethell, 2006). Longer, thinner spines are associated with immature synapses and are highly motile, whereas shorter spines with large spine heads are associated with mature, stable synaptic connections. MMP-9 transgenic overexpression or global bath application of active MMP-9 induces the elongation of dendritic spines, similar to integrin activation, while blocking integrin receptors prevents MMP-9-induced spine alterations (Bilousova et al., 2009; Michaluk et al., 2011). The effects of MMP-9 on integrin receptor signaling are not mediated through direct protein–protein interactions (Michaluk et al., 2009). Rather it is suggested that MMP-9 cleavage of ECM triggers a release of RGD-containing peptides that are potent activators of integrins (Ruoslahti, 1996b). A direct application of RGD-containing peptide to cultured hippocampal neurons was also shown to induce spine elongation and actin reorganization in dendritic spines in an NMDA-dependent manner (Shi and Ethell, 2006), possibly through integrin-mediated phosphorylation of NMDA receptors (Bernard-Trifilo et al., 2005).

Interestingly, local activation of MMP-9 at the level of individual synapses can induce the maturation of spine heads instead of spine elongation. After cLTP in culture, MMP-9 (and possibly MMP-2) enzymatic activity is localized around a small number of immature spines that preferentially undergo spine head enlargement (as opposed to already mature spines; Szepesi et al., 2014). Additionally, experiments using theta burst stimulation to induce LTP in acute hippocampal slices demonstrated that MMP-9 activity is both necessary and sufficient to induce expansion of spine heads, including small and large spines (Wang et al., 2008). Blocking MMP activity produced a transient increase in head size that was quickly reduced to baseline, analogous to the MMP-9 dependent maintenance of LTP (Nagy et al., 2006; Okulski et al., 2007; Wang et al., 2008). Consistent with other reports, MMP-9-mediated spine head enlargement was also dependent on integrin signaling (Wang et al., 2008). In addition, both MMP-9 activation and cLTP were shown to induce the extension of filopodia-like protrusions from the heads of dendritic spines (Szepesi et al., 2013), which were also linked to a local change in connectivity in response to glutamate release (Richards et al., 2005). Taken together this indicates that MMP-9 can induce spine elongation or maturation depending on the level of MMP-9 activity, its localization and/or substrate. Why MMP-9 would have such differential effects is unclear. If endogenous MMP-9 does preferentially target immature spines to induce their maturation in response to neuronal activity (Szepesi et al., 2014), exogenous application of MMP-9 could disrupt this selectivity, targeting mature synapses instead. It is likewise possible that MMP-9 cleavage of specific synaptic and perisynaptic targets at a specific time and place is necessary for spine head enlargement, but that if the action is not temporally restricted MMP-9 activity prevents these synaptic structures from maturing and stabilizing. Indeed, both genetic deletion and overexpression of MMP-9 impairs the maintenance of LTP in the mossy fiber-CA3 pathway in hippocampal slices (Wiera et al., 2013).

Matrix metalloproteinase-9 up-regulation in response to NMDA activity can also act on ICAM-5 and NLG-1 to regulate dendritic spine morphology. ICAM-5 is a cell adhesion molecule found in excitatory cell bodies and in dendritic shafts and spines. In cultured hippocampal neurons, ICAM-5 is found in association with thin spines and filopodia more so than with mature mushroom-shaped spines (Tian et al., 2007; Ning et al., 2013). ICAM-5 within postsynaptic filopodia binds to presynaptic β1integrin receptors to mediate trans-synaptic signaling and this binding maintains immature spine morphology (Ning et al., 2013). Hippocampal neurons treated with NMDA show an increase in mushroom shaped spines with a concomitant increase in ICAM-5 cleavage, indicating that cleavage of ICAM-5 may induce spine maturation (Tian et al., 2007). Both MMP-9 and MMP-2 can cleave ICAM-5, and active levels of both enzymes are elevated after NMDA stimulation (Tian et al., 2007). Likewise, NMDA induced cleavage of ICAM-5 in dendritic shafts is rescued by inhibition of MMP-2 and MMP-9 (Tian et al., 2007). In the visual cortex during a period of active synaptogenesis, just prior to (P14) and during ocular dominance CPP (P28), there is a developmental shift in ICAM-5 localization from dendritic protrusions at P14 to dendritic shafts by P28 (Kelly et al., 2014). However, this shift does not occur in MMP-9 KO mice (Kelly et al., 2014). Instead there are increased ICAM-5 levels in dendritic protrusions at both P14 and P28 which do not change with age (Kelly et al., 2014). An increase in synaptic contacts between ICAM-5 labeled structures (presumably dendritic structures) and axon terminals is also observed in MMP-9 KO mice (Kelly et al., 2013). Together this may indicate that MMP-9 cleaves ICAM-5 in response to NMDA stimulation to induce spine maturation, while in the absence of MMP-9 ICAM-5 remains in dendritic protrusions and maintains immature synapses through its binding to presynaptic β1 integrin receptors. NLG-1 is a postsynaptic adhesion molecule, specific to excitatory cells, which binds to presynaptic neurexins through trans-synaptic interactions (Peixoto et al., 2012). MMP-9 cleaves NLG-1 both *in vivo* and *in vitro*, and this cleavage induces a rapid destabilization of its presynaptic partner neurexin-1 (Peixoto et al., 2012). This destabilization is paralleled by a reduced frequency of mini excitatory postsynaptic potentials and an altered paired pulse ratio – indicative of reduced presynaptic release probability (Peixoto et al., 2012). MMP-9 cleavage of NLG-1 is dependent on NMDA activation and Ca^2+^/calmodulin-dependent protein kinase signaling, and occurs locally at the excited spine (Peixoto et al., 2012). Interestingly, NLG-1 cleavage is also regulated by sensory experience associated with CPP in the visual cortex and is increased within 2 h of light exposure after 5 days of light deprivation (dark rearing) in mice during the fourth postnatal week (Peixoto et al., 2012). Together these studies illustrate a role for MMP-9 in the active modification of postsynaptic sites to modulate synaptic efficacy, a process important both during development and throughout adulthood. However, the role of MMP-9 in presynaptic axon reorganization remains less clear.

### Axon Regeneration and Plasticity

The basic pattern of axonal tract organization is largely established before birth. During embryonic development axonal processes migrate through the developing nervous system relying on chemical cues as well as cell–cell and matrix–cell interactions in order to find their target locations. They then undergo reorganization in response to sensory stimulation during the critical period, where there is an elaboration of axonal arborizations followed by pruning. The axon arbors are thought to remain relatively stable after CPP closure, but can undergo reorganization following injury or sensory deprivation. MMP-2 and MMP-9 have been implicated in embryonic axon path finding in the *Xenopus* (Hehr, 2005) and MMP-9 was more directly implicated in regeneration of axons after injury in the rat (Shubayev and Myers, 2004; Ahmed et al., 2005). MMP-9 protein is also expressed in growing axons during early postnatal synaptogenesis in the hippocampus (Aujla and Huntley, 2014) and is necessary for neurite outgrowth in cerebellar granular cells (Vaillant et al., 2003). Still, the role of MMP-9 in CPP axonal reorganization needs further investigation.

During axon outgrowth and regeneration, MMP-9 activity seems to have differential effects depending on the neuronal type. MMP-9 protein is localized to axon growth cones in regenerating nerve fibers 8 days after sciatic nerve injury in rats (Shubayev and Myers, 2004) and its active enzymatic activity can peak as soon as 21 h after sciatic nerve injury in the mouse (Ferguson, 2000), although this early increase may be associated with inflammatory processes at the injury site rather than with regeneration *per se*. MMP-9 in PC12 cell culture can increase the length of sprouting fibers without affecting the total number of sprouting fibers (Shubayev and Myers, 2004). Similarly, MMP-9 and MMP-2 treatment of dorsal root ganglion neurites grown in culture can increase the average length of the neurites (Ferguson, 2000). Conversely, MMP-9 treatment does not affect either the number or the length of neurites in neonatal spiral ganglion neurons cultured from 5 days old rats (Sung et al., 2014). After olfactory nerve injury, increases in MMP-9 protein levels overlap with degeneration of mature olfactory neurons, while MMP-9 decreases during the period of regeneration (Costanzo et al., 2006). In contrast, both MMP-9 and MMP-2 protein and enzymatic levels are increased in regenerating optic nerves compared to non-regenerating nerves, peaking 8 days after injury (Ahmed et al., 2005).

Mechanisms underlying MMP-9 activity in axon growth and repair are also diverse. MMP-9 and MMP-2 can cleave EphB2, a receptor tyrosine kinase that can differentially attract or repulse axonal fibers through interactions with the ephrin-B ligand (reviewed by Sloniowski and Ethell, 2012). MMP-9 cleavage of EphB2 triggers a cell repulsion and subsequent collapse of axon growth cones in cultured hippocampal neurons through Rho GTPase activation (Lin et al., 2008). Additionally, MMP-9 cleaves CRMP-2 (Bajor et al., 2012), a protein that is shown to induce neuronal polarization and the elongation of axons (Yoshimura et al., 2005) in part through its ability to bind with tubulin heterodimers and to thereby promote microtubule formation (Fukata et al., 2002). How MMP-9 activity might affect the function of CRMP-2 is not yet clear, though the putative cleavage site for MMP-9 of CRMP-2 overlaps with its protein binding site (Bajor et al., 2012), indicating that MMP-9 cleavage might interfere with CRMP-2 binding to tubulin, thereby affecting axonal elongation. Furthermore, MMP-9 can create a permissive environment for nerve regrowth after injury by controlling the proliferation of Schwann cells through induction of the Ras/Raf/MEK–ERK pathway to suspend Schwann cell mitosis (Chattopadhyay and Shubayev, 2009). Together these studies indicate numerous roles for MMP-9 in axonal plasticity, including regeneration and degeneration after injury, polarization and growth of neurite fibers, axon repulsion and growth cone collapse, as well as interactions with glial cells. It remains to be seen whether similar mechanisms play a role in postnatal CNS development and CPP.

### Myelination

Matrix metalloproteinase-9 is also expressed in OL (Oh et al., 1999) and MMP activity is implicated in the regulation of myelin formation in the CNS (Maier et al., 2006). Myelination is one of the last processes to occur in CNS development, a hallmark of mature, established axonal connections. In the CNS, myelination along axonal processes is performed by OL, a type of glial cell. Each oligodendrocyte can myelinate as many as 40 internodes, a process that occurs in several steps. After OL migrate and proliferate during early postnatal development (reviewed by Pfeiffer et al., 1993) three further steps are involved in the process of myelination: (1) OLs begin a period of extensive outgrowth of processes radially from the cell body toward the axons, (2) once contact with an axon has been made, the OL ensheaths the axon in myelin layers until (3) compaction of the myelin sheath occurs (Uhm et al., 1998). Neuronal signals are key in the maturation of OL, where for example adenosine acts as a neuron-glial signal to inhibit the proliferation of OL progenitor cells and promote differentiation and myelin formation (Stevens et al., 2002). Myelin formation peaks within the first 4 weeks after birth (Foran and Peterson, 1992), generally in a caudal to rostral organization (reviewed by Pfeiffer et al., 1993). Axons with larger diameters are myelinated first, followed by smaller-diameter axons (Matthews and Duncan, 1971; Ye et al., 1995b). However, there are specific periods of myelination within different brain regions, each with a specific onset/peak window as well as different organizational trajectories (Foran and Peterson, 1992).

In the mouse optic nerve, MMP-9 enzymatic activity levels gradually increase from P3 to P11, with elevated transcript levels in the optic nerve at P7 (Larsen et al., 2006) and strong gelatinolytic activity at P9 (Oh et al., 1999), a time period overlapping with the strong expression of myelin basic protein (MBP; Oh et al., 1999). In contrast, in the rodent corpus callosum, MMP-9 protein expression increases from P7 to P28 (Uhm et al., 1998), a time that corresponds to developmental myelination. MMP protein and gelatinolytic activity are localized around the OL cell body, along OL processes and, notably, at the advancing tip of OL processes (Oh et al., 1999; Šišková et al., 2009), implicating MMP-9 in the first step of myelination, OL process outgrowth.

Indeed, in mouse, human and bovine cultures of OLs, (Oh et al., 1999) increased OL process formation following activation of PKC is accompanied by MMP-9 secretion, likely the proform of the enzyme, and inhibition of MMP-9 activity reduces OL process outgrowth (Uhm et al., 1998). In MMP-9 KO mice, though the development of myelin formation in the optic nerve is comparable to WT mice, adult OLs in culture have impaired process outgrowth in response to PKC activation and reduced spontaneous process formation (Oh et al., 1999). In the corpus collosum of MMP-9 KO mice, however, there was a transient reduction in the amount of MBP from P7 to P10 and a decrease in the number of mature OL cells (Larsen et al., 2006). The reduction in the number of mature OL cells indicates a role for MMP-9 not only in process extension but also in OL maturation. Šišková et al. (2009) further demonstrated that the interaction of MMP-9 with ECM molecule fibronectin affects the distribution of MMP-9 enzymatic activity along OL processes, restricting its activity to OL cell bodies. This change in MMP-9 localization from control conditions is associated with the inhibition of OL differentiation, characterized by fewer primary and secondary processes, implicating MMP-9 enzymatic activity in OL differentiation (Šišková et al., 2009).

Matrix metalloproteinase-9 may also be involved in the second stage of myelination, ensheathment, through indirect interactions with IGF-1. IGF-1 promotes OL proliferation and/or survival, stimulates myelination production and increases myelin thickness, and can be inhibited by IGFBP-6 (Ye et al., 1995a). MMP-9 and MMP-12 can both cleave IGFBP-6, while MMP-9 null mice show increased levels of IGFBP-6 (Larsen et al., 2006) and application of IGF-1 increases the number of mature OLs in MMP-12 null mice (Larsen et al., 2006). These studies suggest a role for MMP-9 and MMP-12 in cleavage of IGFBP-6 that disinhibits IGF-1 and thereby promotes OL maturation and ensheathment. MMP activity is further implicated in mediating contact between OL cells and axons through a cleavage of the OL-specific 155-kDa isoform of NF155, a member of the L1-family of cell adhesion molecules (L1-CAM; Maier et al., 2006). This function is important because the extracellular domain of NF155, localized on the oligodendrocyte, can interact directly with adhesion molecules located on the axonal membrane, such as contactin (Maier et al., 2006). This suggests a role for MMP-mediated cleavage of NF155 in establishing the interactions between myelin and axons. Thus, MMP-9 is involved in multiple steps of the myelination process including OL maturation, process outgrowth and ensheathment of axons through its association with ECM and neuronal receptors to ‘guide’ the myelination process.

## MMP-9 in Pathophysiology of Neurologic Disorders

While the regulation of MMP-9 is important for many aspects of normal CNS development and plasticity, misregulation of MMP-9 levels and activity is increasingly implicated in neurodevelopmental and psychiatric disorders that are associated with aberrant brain development.

### Fragile X Syndrome and Other Developmental Disorders

Recently, MMP-9 has been implicated in several psychiatric disorders that are associated with abnormal development including FXS, autism spectrum disorder (ASD), bipolar disorder and schizophrenia. Increased levels of MMP-9 have been demonstrated in human subjects with FXS (Dziembowska et al., 2013; Sidhu et al., 2014), ASD (Abdallah et al., 2012), bi-polar disorder (Rybakowski et al., 2013) and treatment-resistant schizophrenia (Yamamori et al., 2013). Still, there is limited literature on the precise function of MMP-9 in these disorders. Whether MMP-9 is responsible for the behavioral deficits associated with these disorders, or is a result of abnormal changes in the brain is still unclear and requires further investigation. However, recent research suggests that enhanced MMP-9 activity is a major factor contributing to the pathophysiology of FXS and epilepsy.

Fragile X syndrome is a trinucleotide repeat disorder that leads to the transcriptional silencing of *Fmr1*. The FMR protein encoded by the gene is involved in translation regulation, and genetic deletion of the *Fmr1* gene in mice mimics FXS-associated deficits, providing a useful model to study FXS. The loss of translational suppression of MMP-9 by FMRP may be driving some of the deficits associated with FXS, such as abnormal dendritic spine development and synaptic plasticity. MMP-9 protein and enzymatic activity levels are increased in the hippocampus of developing (P7), adolescent, and adult *Fmr1 KO* mice (Bilousova et al., 2009; Janusz et al., 2013; Gkogkas et al., 2014; Sidhu et al., 2014). This enhanced MMP-9 activity contributes to abnormal PI3K-Akt-mTOR signaling in the *Fmr1* KO mouse, a pathway implicated in FXS and other ASDs (Sidhu et al., 2014). Moreover, the ability of a tetracycline derivative, minocycline, to inhibit MMP-9 activity is suggested to underlie its beneficial effects in *Fmr1* KO mice. Minocycline treatment lowers MMP-9, but not MMP-2 levels, and restores normal dendritic spine development in young *Fmr1* KO mice (Bilousova et al., 2009), reduces audiogenic seizure severity and rescues abnormal behaviors in both young and adult *Fmr1* KO mice (Rotschafer et al., 2012; Dansie et al., 2013). Genetic deletion of *MMP-9* also repairs abnormal dendritic spine development, normalizes mGluR5-dependent LTD and alleviates abnormal social behaviors and anxiety in *Fmr1* KO mice (Sidhu et al., 2014). In drosophila there are only two MMPs, the secreted form MMP-1 and the membrane-anchored form MMP-2. Inhibition of MMP-1 activity in *dfmr1* null flies, a drosophila model of FXS, rescues some or all of the defects associated with the deletion of *dfmr* (Siller and Broadie, 2011; reviewed by Siller and Broadie, 2012). This was demonstrated using three different approaches: either (1) the overexpression of tissue inhibitor of MMP (TIMP), (2) the generation of double null mutants *dfmr1;mmp1*, or (3) treatment with minocycline (Siller and Broadie, 2011; reviewed by Siller and Broadie, 2012).

In humans with FXS the levels of total MMP-9 enzymatic activity are elevated in the plasma, without changes in MMP-2 levels (Dziembowska et al., 2013). The analysis of postmortem FXS brain tissue samples also indicate elevated levels of MMP-9 protein in both the hippocampus and neocortex (Gkogkas et al., 2014; Sidhu et al., 2014). Minocycline treatment reduces plasma levels of MMP-9 in most but not all subjects, and is associated with improvements in behavior (Paribello et al., 2010; Utari et al., 2010; Dziembowska et al., 2013; Leigh et al., 2013) and a reversal of habituation deficits in auditory event related potentials (Schneider et al., 2013). This suggests that MMP-9 suppression may be a useful therapeutic approach. Together these studies indicate that increased MMP-9 levels in FXS may underlie molecular, cellular and behavioral deficits, many of which are associated with abnormal plasticity, learning and memory in FXS.

### MMP-9 in Epilepsy

The role of MMP-9 in epilepsy has been seen in both humans (Suenaga et al., 2008; Konopka et al., 2013) and in animals, and is a useful model of MMP-9 in aberrant plasticity. Epilepsy is a disorder in which seizures become spontaneous and recurrent, with highly synchronized activity. Aberrant neuronal plasticity as a result of seizure activity or brain injury is thought to underlie the progression of epilepsy. In both animal models of epilepsy and human epilepsy subjects (Proper et al., 2000) this aberrant plasticity can include neuronal loss in hippocampal regions, astrogliosis, aberrant pruning of DG spines (Wilczynski et al., 2008), reorganization of interneuron terminals (Pollock et al., 2014), loss and/or disorganized PNN structures (McRae et al., 2012; Pollock et al., 2014), and the sprouting of mossy fibers to create a recurrent network (Wilczynski et al., 2008).

In young and adult human epilepsy patients MMP-9 protein levels are higher compared to controls (Konopka et al., 2013). Though other MMPs are increased as well, including MMP-2, MMP-3 and TIMP-2 in adults, MMP-9 has the most prominent and consistent increase in protein levels across ages (Konopka et al., 2013). This protein expression is localized to neurons, astrocytic processes and synapses, and is more strongly expressed in dysmorphic neurons than healthy neurons (Konopka et al., 2013). One mechanism thought to underlie epilepsy progression may be through a change in the ratio of MMP-9 to TIMP-1, the endogenous inhibitor of MMP-9. Subjects with varying forms of epilepsy have an increase in the MMP-9/TIMP-1 ratio suggesting an increase in the extracellular levels of MMP-9 activity (Suenaga et al., 2008).

In rodent models of epilepsy, a kindling procedure can be used where animals are electrically or chemically stimulated at sub-threshold seizure levels over a period of weeks until spontaneous seizures take place. MMP-9 KO mice require a longer kindling period to develop epilepsy over controls (Wilczynski et al., 2008; Mizoguchi et al., 2011), and experience less severe seizures once fully kindled and an increased survival rate (Wilczynski et al., 2008). Conversely, transgenic MMP-9 overexpressing rats show increased susceptibility to epileptogenesis with kindling (Wilczynski et al., 2008). After epilepsy induction in mice, overall MMP-9 protein levels did not change in the DG; however, there was a marked increase in the number of spines expressing MMP-9 protein and in gelatinolytic activity localized to dendrites (Wilczynski et al., 2008). Interestingly, DG spine density was significantly decreased in WT but not MMP-9 KO mice after kainite-induced seizure indicating MMP-9 may play a destructive role promoting kainate-evoked spine pruning (Wilczynski et al., 2008). The inhibition of MMP-9 in slices after kainate application produces a 90% decrease in the density of mossy fiber sprouting in the DG (Wilczynski et al., 2008). This indicates a functional role for MMP-9 both in synaptic pruning and in mossy fiber sprouting in rodent models of epilepsy.

It is likely that there are multiple targets of MMP-9 which contribute to the progression of epilepsy. It is possible that MMP-9 acts by creating a permissive state through cleavage of ECM molecules, in particular, through cleavage of aggrecan. After seizure induction there is a loss of PNNs around parvalbumin positive interneurons (Pollock et al., 2014), including a specific reduction in aggrecan and an increase in unbound hyaluronan (McRae et al., 2012). Doxycycline hyclate, an antibiotic that inhibits MMP-9, prevents the loss of PNNs after kindling and delays epilepsy onset, possibly through the inhibition of MMP-9 (Pollock et al., 2014). MMP-9 mediated cleavage of ECM may also affect neuronal activity and potentiate NMDAR currents through the activation of integrins (see Synapse Development and Plasticity for details). In addition, MMP-9 cleavage of pro-BDNF into mature BDNF may play a role in the development of epilepsy through mossy fiber sprouting. The BDNF to pro-BDNF ratio increases in kindled WT mice, but not in MMP-9 KO mice, during the progression of epilepsy (Mizoguchi et al., 2011) implicating MMP-9 in pro-BDNF cleavage. Because there is no difference in overall BDNF levels in fully kindled mice, this suggests that MMP-9 cleavage of pro-BDNF may accelerate the onset of epilepsy (Mizoguchi et al., 2011).

### Therapeutic Approaches

There are several drugs on the market that can directly or indirectly alter MMP-9 levels and activity, some of which are already in use in humans. For example, diazepam, a drug used to treat epilepsy in humans, is able to suppress the development of epilepsy in kindled mice and reduce MMP-9 levels (Mizoguchi et al., 2011). Likewise both doxycycline (Pollock et al., 2014) and minocycline (Beheshti Nasr et al., 2013; Dansie et al., 2013) treatment can reduce seizure severity or epilepsy onset in mice. Minocycline treatment also rescues behavioral, anatomical and social deficits in young and adult *Fmr1* KO mice (Bilousova et al., 2009; Rotschafer et al., 2012; Dansie et al., 2013). Clinical trials on the effects of minocycline in human subjects with FXS have demonstrated improvement in language, social communication, anxiety and attention (Utari et al., 2010) stereotypy, irritability and hyperactivity (Paribello et al., 2010) as well as on behavioral-improvement scales based on caregiver reports in a randomized double-blind, placebo-controlled trial in children and adolescents with FXS (Dziembowska et al., 2013; Leigh et al., 2013). What’s more, electrophysiological measures in the auditory cortex of human subjects with FXS indicate a reduction in auditory hyper-reactivity with minocycline treatment (Schneider et al., 2013).

Considering the widespread action of minocycline in the CNS, the effects of minocycline in FXS may not be limited to the inhibition of MMP-9. Minocycline can inhibit other MMPs and is shown to have anti-inflammatory and anti-apoptotic effects (reviewed by Elewa et al., 2006), can phosphorylate AMPA receptors (Imbesi et al., 2008), and affect MAPK signaling (Nikodemova et al., 2006). Doxycycline similarly has various effects on CNS functions. On the other hand, MMP-9 is itself expressed by many types of tissue throughout the body and, as in the CNS, it can have both detrimental and beneficial effects on biological systems (reviewed by Fingleton, 2008). The potential side effects of non-specific MMP-9 inhibition to treat neurodevelopmental disorders have not been thoroughly investigated, though MMP-9 KO mice have shown detrimental side effects as well as benefits in animal models of cancer (Coussens et al., 2000) and kidney disease (Zeisberg et al., 2006) and highlight the need for treatments specific to both the CNS and to key points in the progression of a disorder. Nonetheless recent studies discussed herein do demonstrate a relationship between the ability of minocycline to inhibit MMP-9 in FXS and a concomitant improvement on behavioral, morphologic and physiological levels. Side effects of the treatment as reported in these studies are minor and are commonly associated with gastrointestinal discomfort (Paribello et al., 2010; Utari et al., 2010; Dziembowska et al., 2013). Altogether, this body of research points to the need for additional studies to understand the specific role of MMP-9 in the etiology of a disorder, including the time frame during which MMP-9 activity may have the largest deleterious effects, and to develop treatments targeted to MMP-9 in the progression of each disorder to further improve the lives of people with intellectual disabilities and autism.

## Conclusion

Untangling the precise functions of MMP-9 is challenging. This is due to the fact that it is expressed by both neurons and glia; that its secretion and activation are regulated by a wide variety of factors; and that it can act on multiple targets, each of which can have differential and even opposing effects. In this review, we summarized the various roles of MMP-9 during early postnatal development within the CNS, with a focus on sensory development.

These developmental events, many of which recruit MMP-9 activity, occur in an organized and interactive fashion through specific cellular targets and in response to sensory experience that drives neuronal activity. MMP-9 recruited to specific extracellular sites can cleave ECM creating room within the extracellular space for cells to migrate and for neuronal processes to grow. Yet beyond being simply permissive, MMP-9, through cleavage and/or activation of cell surface and cell adhesion molecules, is implicated in active dendritic spine remodeling and stabilization; pre- and postsynaptic receptor dynamics; consolidation of LTP; myelination and synaptic pruning. It is further implicated in axonal sprouting, path finding, regeneration and degeneration. MMP-9 is expressed just prior to and during CPP, a peak of reorganization in the sensory cortex, and its activity is down regulated in the adult brain. However, unregulated activity of MMP-9 can have detrimental effects on brain functions and may underlie deficits observed in several neurodevelopmental disorders. It is therefore important to better understand the mechanisms by which MMP-9 mediates early postnatal development and its role in neurodevelopmental disorders.

Several key questions remain unanswered. The role of MMP-9 in auditory CPP, for instance, is yet to be studied. Tonotopic maps in rodent primary auditory cortex do undergo reorganization during a ‘sensitive window’ after which the network is less plastic. Reorganization of the tonotopic frequency map can take place with as few as 3 days exposure to a single tone, from P11 to P13 (de Villers-Sidani et al., 2007), though time windows used to test auditory CPP vary (Webster, 1983; Zhang et al., 2002; Barkat et al., 2011; Kim et al., 2013). Recent studies have demonstrated an auditory CPP deficit in *Fmr1* KO mice (Kim et al., 2013) that may be related to enhanced levels of MMP-9 activity in the brain. The role of MMP-9 in these auditory deficits remains unclear. Because clarification may lead to new therapeutic strategies to treat auditory hypersensitivity and habituation deficits seen in FXS and ASD, understating this role is important.

Though MMP-9 seems to consistently be associated with early postnatal development across many sensory regions and is downregulated by adolescence and into adulthood, mechanistic studies of MMP-9 have largely focused on l-LTP and synaptic plasticity in cultured hippocampal neurons. Furthermore, it is known that during development many receptors undergo changes in sub-receptor composition, localization and/or distribution within a network, as well as in their physiological properties. Similarly, intracellular signaling molecules, as well as cell surface and cell adhesion molecules and ECM components all have altered expression throughout development. Therefore it is uncertain whether the mechanisms underlying MMP-9 recruitment and activity in adolescent and adult hippocampal neurons are conserved across cortical regions throughout development.

In addition, given the overexpression of MMP-9 in multiple neurodevelopmental disorders, it is important to understand whether MMP-9 plays a regulatory and/or permissive role in critical period development, specifically through its interactions with ECM. The formation of PNN structures in the CNS, a specific sub-type of ECM, has been linked to the closure of visual CPP in rodents (reviewed by Hensch, 2005), associated with developmental song learning in the zebra finch (Balmer et al., 2009), and has been shown to be altered with sensory deprivation in rodent barrel cortex (McRae et al., 2007). MMP-9 can directly or indirectly induce cleavage of multiple components of PNN structures, including laminin, brevican, tenascin-R, and aggrecan (reviewed by Ethell and Ethell, 2007). Therefore elevated levels of MMP-9 during development may induce aberrant cleavage and alterations in PNN structures. It remains to be seen whether PNNs are indeed altered in neurodevelopmental disorders and whether MMP-9 inhibition can recover normal CPP through the stabilization of PNNs.

While MMP-9 activity is important for normal CNS development and for the consolidation of long-term memories from development through adulthood, misregulated expression and/or activity of MMP-9 is associated with many neurological disorders. Therefore, understanding the action of MMP-9 within specific cortical regions, developmental periods and in specific cellular processes is important for providing groundwork for new therapies that target MMP-9 in treatments for neurodevelopmental and other disorders.

## Conflict of Interest Statement

The authors declare that the research was conducted in the absence of any commercial or financial relationships that could be construed as a potential conflict of interest.

## Abbreviations

CNS: central nervous system
CPP: critical period plasticity
CRMP-2: collapsin response mediator protein-2
DG: dentate gyrus
ECM: extracellular matrix
EGL: external granular layer
e-LTP: early-phase long term potentiation
FXS: Fragile X syndrome
*Fmr 1*: Fragile X mental retardation gene 1
FMRP: Fragile X mental retardation protein
ICAM-5: intercellular adhesion molecule-5
IGF-1: insulin growth factor – 1 (IGF-1)
IGBP-6: insulin growth factor binding protein – 6
IGL: internal granular layer
L1CAM: L1 cell adhesion molecule
l-LTP: late-phase long term potentiation
MAPK: mitogen-activated protein kinase
MMP-9: matrix metalloproteinase-9
NF155: neurofascin
NLG-1: neuroligin-1
OL: oligodendrocytes
PKC: protein kinase C
PNN: perineuronal net
RGD: arginine-glycine-aspartate
SC: superior colliculus
TIMP-1: tissue inhibitor of metalloproteinase-1.
